# Contraceptive considerations for breastfeeding women within Jewish law

**DOI:** 10.1186/1746-4358-2-1

**Published:** 2007-01-04

**Authors:** Ilana R Chertok, Deena R Zimmerman

**Affiliations:** 1 West Virginia University School of Nursing, Morgantown, West Virginia, USA; 2 University of Illinois at Chicago, College of Nursing, Chicago, Illinois, USA; 3 TEREM Immediate Medical Services, Israel; 4 Nishmat: The Jerusalem Center for Advanced Jewish Study for Women, Jerusalem, Israel

## Abstract

Breast milk has been shown to have multiple benefits to infant health and development. Therefore, it is important that maternal contraceptive choices consider the effects on lactation. Women who observe traditional Jewish law, halakha, have additional considerations in deciding the order of preference of contraceptive methods due to religious concerns including the use of barrier and spermicidal methods. In addition, uterine bleeding, a common side effect of hormonal methods and IUD, can have a major impact on the quality of intimacy and marital life due to the laws of *niddah*. This body of Jewish laws prohibits any physical contact from the onset of uterine bleeding until its cessation and for an additional week. Health care professionals should understand the issues of Jewish law involved in modern contraceptive methods in order to work in tandem with the halakha observant woman to choose a contraceptive method that preserves the important breastfeeding relationship with her infant and minimizes a negative impact on intimacy with her husband.

## Review

### Medical encouragement of breastfeeding

Breastfeeding is endorsed by major health organizations as the optimal form of infant nutrition. When infants do not receive breast milk, there are increased health risks for both mother and infant. For women who do not breastfeed, there are increased risks such as an increased risk of breast cancer [1,2]. Research in developed and developing countries has demonstrated that infants who are not breastfed are at increased risk of infant mortality [3-5]. Infants who do not receive breast milk have an increased incidence of many acute [5,6] and chronic conditions [7-9]. To quote the recent policy statement of the American Academy of Pediatrics, "Human milk is species-specific, and all substitute feeding preparations differ markedly from it, making human milk uniquely superior for infant feeding" [10] (see p. 496). Exclusive breastfeeding is the ideal infant nutrition; it is sufficient to support optimal growth and development for approximately the first six months of life. This half-year of exclusive breastfeeding should be followed by a gradual introduction of iron-enriched complementary solid foods with continued breastfeeding for at least twelve months, and thereafter for as long as mutually desired. Therefore, it is important for all health care professionals to protect breastfeeding and minimize barriers that may threaten the mother-infant breastfeeding relationship.

### Jewish law and its place within Judaism

The composite body of Jewish law is known as halakha. It is a system that has evolved over at least two millennia. Written halakhic works include the *Mishna *(edited approximately 200 A.D.), the *Talmud *(edited approximately 500 A.D.), and the works of Maimonides (written in approximately 1250 A.D.). The *Shulkhan Arukh *(written in 1564 by Rabbi Yosef Karo) is considered a binding codification of laws with discussion and development via response, rabbinic questions and answers, continuing to the present day. Questions that arise within or related to Jewish law are addressed to a halakhic authority for clarification of proper practice. The laws subsumed under halakha may be divided into biblical and rabbinic origin. Both are binding on the halakha observant Jew. However, at times rabbinic injunctions have more room for leniency, especially in extenuating circumstances. In general, people who ascribe themselves to Orthodox Judaism view halakha as binding. The proportion of Jews who identify themselves as Orthodox varies with location. Even within those who define themselves as Orthodox there is variation in the strictness of practice and the degree to which they consult with a rabbinic authority when questions of practice arise.

### Encouragement of breastfeeding and childbearing within Jewish law

Breastfeeding is valued by Jewish tradition, as are health promotional practices [11]. The *Talmud *discusses breastfeeding duration in a number of contexts and in most cases assumes a duration of 24 months (*Ketuvot *60a, b) [12]. The *Shulkhan Arukh *codifies a minimum of two years (*Even Haezer *143:8) and a maximum of five years (*Yore Deah *81:7) [13]. Maimonides recommends breastfeeding in his compilation of Jewish law, the *Mishneh Torah LeRambam *(*Gerushin *11:26) [14]. There are other references in traditional texts including discussion in the *Talmud *regarding breastfeeding durations of two through five years [15]. There is also mention of the importance of preserving breastfeeding that could potentially be challenged by subsequent pregnancy (*Yevamot *12b) [12].

Jewish tradition values childbearing, as derived from the biblical commandment and divine blessing in Genesis (1:28 and 9:7), "be fruitful and multiply" [16]. Maimonides explains that this commandment is considered fulfilled with the births of at least one son and one daughter and indicates a separate rabbinic injunction to continue having children as long as one is able. (*Mishneh Torah LeRambam, Hilchot Ishut*, chapter 15) [14]. The biblical commandment to procreate is considered to be directed to men, with women being the conduit through which this commandment is fulfilled.

### Permissibility of contraception

Despite the halakhic emphasis on childbearing, there are times where contraception is permitted or even mandated. Permission for use of contraception is carefully considered for each individual couple by a halakhic authority. The most halakhically endorsed indication for contraceptive use is when pregnancy poses a threat to a woman's life related to the Jewish principle of *Pikuach Nefesh*, saving a life, whereby danger to life suspends all commandments, excluding adultery, idolatry and murder (*Shulkhan Arukh, Yore Deah *336:1) [13]. Contraception may be considered under other situations as well, where a woman's physical or emotional well-being is threatened [17-19], and in the case of the breastfeeding maternal-infant dyad, to preserve the provision of breast milk quality and quantity for the infant, as stated in the Talmud (*Yevamot *12b) [12,20]. However, there are halakhic considerations and issues for both men and women that must be taken into account in determining the method of contraception. Overall, it is easier to permit female contraceptive use rather than male contraceptive use as the nature of the injunction not to use male contraception is biblical and therefore considered of a higher order compared to the rabbinic injunction regarding female contraceptive use [17].

### Talmudic discussion of female contraception

Halakhic considerations often begin with Talmudic discussion and precedents. One method of female contraception mentioned in the *Talmud *is the *moch*, defined as either a tampon-like device inserted into the vagina prior to intercourse or a post-coital sponge. Some contemporary halakhic authorities compare the *moch *to modern barrier methods [15] such as the diaphragm, cervical cap and contraceptive sponge. The *Talmud *(*Yevamot *12b) [12] discusses use of the *moch *as a contraceptive method by lactating women, although the extent and conditions of its use are debated. These debates play a role in the contemporary rabbinic hesitation in permitting the use of barrier methods [21]. Another method mentioned in the *Talmud *is a contraceptive drink used to induce infertility that may have been permitted for women with a history of traumatic childbirth [15].

### Halakhic considerations in choice of contraceptive method

Permanent sterilization (s*irus*) is prohibited based upon a biblical prohibition (Leviticus 22:24) [16], which is considered for men of strict (biblical) origin, whereas, for women, of less strict (rabbinic) decree, as delineated in the halakhic work, the *Shulkhan Arukh *[13]. As such, vasectomy and tubal ligation, considered permanent contraception, are prohibited by Jewish law. There is some leniency for tubal ligation in special cases, especially since this method may be reversed or circumnavigated with assisted reproductive technology [22-24].

Another important consideration for contraceptive use is the halakhic prohibition of purposeful emission of semen outside the vaginal canal. Therefore, coitus interruptus is forbidden (*Yevamot *34b) [12]. Likewise, condom use is forbidden since the condom impedes sperm from entering the vaginal canal. This issue has implications for female use of barrier methods as well, as will be further discussed. As the currently available male methods of contraception, condoms and vasectomy, are prohibited, the remaining discussion is limited to female methods of contraception.

A critical consideration regarding female use of contraception is the propensity of the method to cause uterine bleeding or staining. According to Jewish law, a woman is rendered a *niddah *when she experiences uterine spotting or bleeding that is not due to injury. While she is a *niddah*, no physical contact is permitted between husband and wife, thereby affecting marital intimacy. A woman who has entered the status of *niddah *remains in that state until she has completed the ritual requirements regardless of how many days she actually bleeds. These requirements include a minimum of twelve days that must elapse from the onset of her *niddah *status and proper immersion in a ritual bath. The twelve day minimum is divided into two phases: (1) a five day minimum for menstruation, even if her menses are shorter than five days; and (2) seven "clean" days, whereby the woman verifies that bleeding has not recurred by doing internal self examinations. Any intermittent bleeding during this process may necessitate restarting the count of seven days. A woman after childbirth must also follow the laws of *niddah *and refrain from physical contact with her husband until all postpartum bleeding has stopped and the additional week of the "clean" days has passed. Likewise, bleeding or spotting that may accompany some contraceptive methods (and are often considered medically insignificant) may significantly impact the intimacy and quality of marital life for women who observe the Jewish laws pertaining to *niddah*. Since not every stain renders a woman a *niddah*, a woman experiencing breakthrough bleeding should consult with her halakhic authority. For halakhic questions regarding the issues of family purity and to learn more about women's health issues in Jewish law, refer to online website resources (including [25] and [26]), or contact the telephone hotline 1-877-YOETZET (toll free in the United States and Canada).

In general, when contraception is used, it should be a method that does not involve male awareness of its use during intercourse and should not interfere in normal sexual relations. The following discussion will address the halakhic considerations of specific contraceptive methods for women.

### Non-artificial methods of contraception

For the lactating woman, a short-term method of contraception that poses no halakhic problem is that of the lactational amenorrhea method (LAM). This method, as described in the medical literature, capitalizes on the anovulation associated with breastfeeding for up to six months postpartum, assuming the woman has not resumed menses and is exclusively or almost exclusively breastfeeding (including breastfeeding at night). LAM has been demonstrated to be over 98% effective in trials worldwide [27,28]. It also has the added health benefits for mother and infant. Halakha encourages breastfeeding duration of at least two years, without concern for potential reduction in fecundity during lactation [29]. LAM poses no halakhic problem and thus should be encouraged for breastfeeding women who are not considering artificial methods, allowing the woman time for postpartum recuperation. Practically, however, for women who require highly effective and reliable contraception, LAM may be insufficient. While some women experience lactational amenorrhea as long as they breastfeed, they cannot be assured of effective prevention of pregnancy beyond the first six months. Furthermore, the method is based on frequency of breastfeeding, including night feeds. For women who leave their infants and work outside the home, the difficulties in maintaining this frequency can increase the failure rate [27].

Natural family planning (NFM) is another non-intervention method involving the measurement of physiologic changes, including basal body temperature and cervical mucous changes [30]. In the lactating woman, these physiologic changes are often difficult to detect. Obtaining basal temperature changes requires at least four hours of uninterrupted sleep, which may be challenging for the breastfeeding mother. Unlike LAM, where intercourse can take place whenever desired by the couple, intercourse must be avoided (or combined with a barrier method) at the time that the woman is potentially fertile, which requires halakhic permission. Furthermore, as the halakha observant woman may not have physical contact with her husband for at least the first twelve days of her cycle, removing additional days until after the fertile period has passed increases the challenge to the intimate life of the couple.

### Artificial methods of contraception: spermicide

For women who decide to use artificial means of contraception, there are various options, which include spermicide, barrier methods, intrauterine device and hormonal preparations. Spermicide, usually containing varying concentrations of nonoxynol-9, destroys sperm on contact. Higher concentrations of spermicide, such as in vaginal suppository form, are 90% effective in preventing pregnancy as compared to the effectiveness of spermicide gels and films (ranging from 78%–88% in the six month study period) [31]. This method is not deleterious to the breastfeeding mother-infant dyad, as it does not enter the milk nor affect production. Most halakhic authorities permit use of spermicide when contraception is indicated since its use alone does not impede vaginal intercourse [32]. Additionally, halakhic authorities consider it a temporary method of contraception with a method of action that is activated only after emission of sperm into the vaginal canal [15]. Use of spermicide does not usually pose bleeding risks to women, although women who are sensitive to spermicide may experience vaginal irritation and bleeding. This bleeding, of vaginal and not of uterine origin, may cause concern to the halakha observant woman, as it may be confused with uterine bleeding. This problem can often be addressed by the use of a different brand or formulation of spermicide, or by considering a different contraceptive method.

### Artificial methods of contraception: barrier methods

Barrier methods such as the diaphragm, cervical cap, and contraceptive sponge are more effective with use of spermicides and may be more effective than spermicide use alone. These methods are not deleterious to the breastfeeding mother-infant dyad as they do not have a systemic effect on the mother and therefore do not affect the quality or quantity of milk. However, they are often considered a less preferred option for the halakha observant woman [21]. This is due to the fact that some halakhic authorities consider the diaphragm analogous to the Talmudic *moch *whose use is limited. Additionally, the diaphragm impedes the progress of the sperm to the cervix, although it does not prevent the deposit of the sperm into the vaginal canal. Nevertheless, some halakhic authorities permit the use of the diaphragm or cervical cap since proper insertion is deep enough for the husband to be unaware of its presence and intercourse continues to be performed in a natural manner. Compared to the diaphragm and cervical cap, contraceptive sponges are less halakhically desirable since they occupy a large area of the vagina, potentially impeding normal intercourse; however there are halakhic authorities who permit their use.

### Artificial methods of contraception: intrauterine device

The intrauterine device (IUD) is a reversible contraceptive method whose mechanism of action is not completely clear and does not disrupt breastfeeding or milk production. Likewise, the IUD containing the progestin levonorgestrel (e.g. Mirena) should not disrupt breastfeeding or milk production [33]. Since the IUD is reversible and does not interfere with sexual relations, it should be a halakhically accepted form of contraception. However, there is some debate. Some rabbinic authorities permit use of the IUD based on the understanding that its mechanism of action is interference with implantation, while others consider the IUD to be a form of abortion and prohibit its use [34]. Furthermore, there are health issues that have raised concern with the IUD, such as the associated risk of pelvic inflammatory disease [35], while recent research has minimized some concerns such as tubal infertility [36]. Since preserving health is of concern in Jewish law and the health risks have been demonstrated, some halakhic authorities do not support use of this method. Another potential problem of the IUD for the halakha observant woman is the increased risk of bleeding and staining for months following insertion and increased risk of extended *niddah *status.

### Artificial methods of contraception: hormonal preparations

There are two categories of contraceptive preparations. One category is progesterone-only, available in oral ("the mini pill"), injectable (e.g. Depo-Provera), and subcutaneous implant (e.g. Norplant) forms. The other contains combinations of estrogen and progesterone available as a pill, patch and vaginal ring. Hormonal methods which include estrogen are contraindicated in women with medical conditions such as liver disease, elevated blood pressure and clotting problems.

Hormonal contraception is often discouraged for breastfeeding women. The two concerns expressed are the transfer of hormones to the infant via the breast milk and the effect on milk quality and/or quantity. The transfer of hormones via human milk is minimal and no long-term adverse effects on child growth and development have been reported. The American Academy of Pediatrics considers hormonal contraceptives to be compatible with breastfeeding [37]. Regarding the effect on milk, progesterone-only methods do not diminish the volume or composition of breast milk [38], thus they are the preferred method of hormonal contraception for the lactating woman. Out of concern for protecting the normal drop in progesterone following delivery of the placenta, necessary for successful lactation, progesterone-only contraception should not be started prior to three weeks postpartum [38]. On the other hand, there is a risk of decreased milk volume with use of combined hormonal contraceptives [39]. For the breastfeeding woman, use of combined hormonal contraceptive should be discouraged or at least delayed until six months postpartum so as not to disturb exclusive breastfeeding. If a breastfeeding woman should decide to use combined hormonal preparations within the first six months, she should be advised to wait until breast milk production is well established, be given anticipatory guidance regarding methods of maintaining and increasing milk supply, and started on the lowest possible dosage of estrogen.

According to most halakhic opinions, hormonal regulation is the first choice of medical contraception; it is temporary and provides no physical barrier to intercourse. Some authorities, however, are concerned about the rare but serious side-effects associated with the estrogen containing methods including increased risk of thromboembolic events. Unpredictable vaginal bleeding is a common complaint with progesterone-only methods [40], an issue of marked concern to the halakha observant woman. The problem can be compounded with the injectable form since the drug remains in the body for an extended period following injection and the symptoms may take months to resolve. Similarly, the subcutaneous implants require surgical removal for resolution of the undesired symptoms. With the combination methods as well there is an increased risk of extended *niddah *status since breakthrough bleeding can be a concern, especially in the first few months [41,42].

## Conclusion

Breastfeeding women should choose methods that are not deleterious to the success of lactation or to the infant's health. The halakha observant woman is faced with a challenge regarding contraceptive use during lactation. The order of preference for the halakha observant breastfeeding woman may vary from the medical order of preference. Following halakhic permission to use artificial contraception, halakhic preference includes methods that are safe, effective, do not interfere with normal sexual relations, and have minimal side effects, especially intermenstrual bleeding or staining. It is common for the halakhic authority to advise hormonal contraception, assuming no contraindications. It is important for a woman who had experienced side effects with this method, especially bleeding or staining, to re-consult with her halakhic authority.

When hormonal preparations are chosen for contraception during lactation, the preference is a progesterone-only method since preparations containing estrogens may threaten breastfeeding success. Switching contraceptive methods may be recommended if intermenstrual bleeding or staining is experienced, rendering the woman a *niddah*. Subsequently, or alternatively, an IUD may be recommended. This can also pose problems of bleeding and staining placing stress on intimacy. Female barrier methods and spermicides are safe for the breastfeeding mother-infant dyad and do not pose complications such as bleeding and staining, although they are not a simple issue for the halakha observant woman.

Knowledge and sensitivity of the many factors that may influence the choice of contraceptive means is necessary for providing each individual woman with appropriate advice regarding contraception during lactation. Dual consultation with the health care provider and halakhic authority should help the lactating halakha observant woman choose a contraceptive method that preserves the important breastfeeding relationship with her infant.

## Competing interests

Dr. Ilana Chertok declares that she has no competing interests. Dr. Deena Zimmerman is the director of the websites run by Nishmat, the Jerusalem Center for Advanced Jewish Studies for Women, on the topic of women's health and Jewish law and is the medical advisor for Nishmat's Golda Koschitzky Women's Halakhic Hotline.

## Authors' contributions

Both authors contributed to the research of the material and preparation of the manuscript.
